# Understanding TAVR device expansion as it relates to morphology of the bicuspid aortic valve: A simulation study

**DOI:** 10.1371/journal.pone.0251579

**Published:** 2021-05-17

**Authors:** Jonathan Kusner, Giulia Luraghi, Farhan Khodaee, José Félix Rodriguez Matas, Francesco Migliavacca, Elazer R. Edelman, Farhad R. Nezami

**Affiliations:** 1 Harvard Medical School, Boston, MA, United States of America; 2 Laboratory of Biological Structure Mechanics (LaBS), Department of Chemistry, Materials and Chemical Engineering ‘Giulio Natta’, Politecnico di Milano, Milan, Italy; 3 Institute for Medical Engineering and Science, Massachusetts Institute of Technology, Cambridge, MA, United States of America; 4 Cardiovascular Division, Brigham and Women’s Hospital, Harvard Medical School, Boston, MA, United States of America; 5 Thoracic and Cardiac Surgery Division, Brigham and Women’s Hospital, Harvard Medical School, Boston, MA, United States of America; Bern University Hospital, SWITZERLAND

## Abstract

The bicuspid aortic valve (BAV) is a common and heterogeneous congenital heart abnormality that is often complicated by aortic stenosis. Although initially developed for tricuspid aortic valves (TAV), transcatheter aortic valve replacement (TAVR) devices are increasingly applied to the treatment of BAV stenosis. It is known that patient-device relationship between TAVR and BAV are not equivalent to those observed in TAV but the nature of these differences are not well understood. We sought to better understand the patient-device relationships between TAVR devices and the two most common morphologies of BAV. We performed finite element simulation of TAVR deployment into three cases of idealized aortic anatomies (TAV, Sievers 0 BAV, Sievers 1 BAV), derived from patient-specific measurements. Valve leaflet von Mises stress at the aortic commissures differed by valve configuration over a ten-fold range (TAV: 0.55 MPa, Sievers 0: 6.64 MPa, and Sievers 1: 4.19 MPa). First principle stress on the aortic wall was greater in Sievers 1 (0.316 MPa) and Sievers 0 BAV (0.137 MPa) compared to TAV (0.056 MPa). TAVR placement in Sievers 1 BAV demonstrated significant device asymmetric alignment, with 1.09 mm of displacement between the center of the device measured at the annulus and at the leaflet free edge. This orifice displacement was marginal in TAV (0.33 mm) and even lower in Sievers 0 BAV (0.23 mm). BAV TAVR, depending on the subtype involved, may encounter disparate combinations of device under expansion and asymmetry compared to TAV deployment. Understanding the impacts of BAV morphology on patient-device relationships can help improve device selection, patient eligibility, and the overall safety of TAVR in BAV.

## Introduction

The bicuspid aortic valve (BAV) is the most common congenital heart defect with an estimated prevalence of 1.3% in the United States, representing approximately 4.3 million individuals [1]. BAV is associated with many secondary complications which together present a massive disease burden. One in three individuals with BAV are expected to develop moderate to severe aortic stenosis (AS) in their lifetime [2]. Transcatheter Aortic Valve Replacement (TAVR) has emerged, after some trepidation, as showing promise in the treatment of BAV AS.

There are several means of classifying the different morphologies of BAV. One of the most widely used systems, the Sievers classification system, uses the number of raphe to characterize BAV subtypes. Yet, few reports investigating BAV TAVR have stratified BAV outcomes and complications according to any classification system. Although it has been reported that patient-device relationships are substantially different between devices deployed in BAV compared to those deployed in tricuspid aortic valves (TAV), it is not known how TAVR deploys within the different morphologies of BAV and how such patient-device relationships may lend insight into the mechanism of BAV TAVR complications [3].

TAVR deployment in BAV has been recognized as a challenging procedure, the performance of which may be enhanced by computer simulation [4]. Computer simulation has been used clinically for BAV TAVR to optimize sizing and implantation procedures, predict flow complications such as paravalvular leak (PVL), and retrospectively determine risk markers for conduction abnormalities [5,6]. Prospective computational analysis of BAV TAVR has found its greatest application in predicting the risk, severity, and location of PVL [6–8].

Although prospective simulations of BAV TAVR represent a leap forward in both the management of BAV and the application of medical computer simulation to clinical practice, the current resource demands of these techniques make their availability limited to select patients at a limited number of clinical centers. Although previous reports have included anatomic subclassification of BAV, stratification of TAVR complications and prospective risk by valvular morphology is still emerging. Identifying and understanding anatomic risk markers that can be evaluated on routine testing, may subvert the need for full patient-specific computational simulation of BAV TAVR, improving the safety of this technology for all BAV patients regardless of institutional patient volume and computational resources.

We performed in silico modeling of TAVR deployment within TAV and the two most common morphologies of BAV. We hypothesized that TAVR device expansion and force distribution would differ with different BAV morphologies and that such differences may be greater between BAV morphologies than differences observed between any given BAV and TAV. Our findings indeed support that BAV cannot be considered as a single entity. Consideration of TAVR for BAV should take into account BAV morphology, relative positions of annular and orifice anatomy, and influence of the eventual flow pattern, all of which may distinguish risks and approaches in the TAVR era.

## Methods

### BAV and TAV geometries

**Fig 1:**
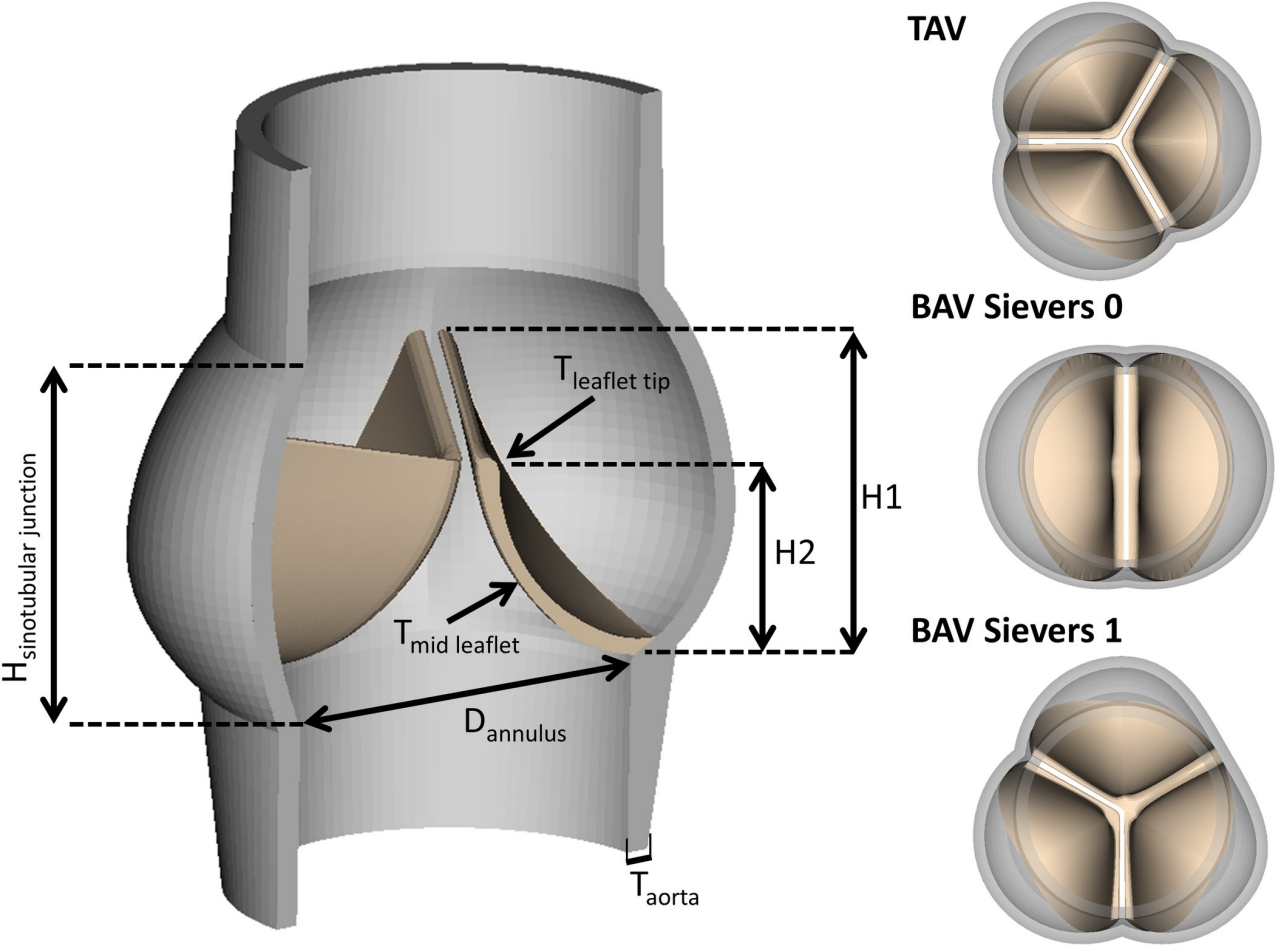
In silico anatomic models of TAV, BAV Sievers 0, and BAV Sievers 1. Labelled values are retained throughout all valve models. H_sinotubular junction_ = 22.1 mm, H1 = 18 mm, H2 = 14 mm, D_annulus_ = 25 mm, T_aorta_ = 1.5 mm, T_mid leaflet_ = 0.8 mm, T_leaflet tup_ = 1.3 mm. Both TAV and BAV Sievers 1 demonstrate commissures that span 120 degrees while BAV Sievers 0 commissures are placed 180 degrees apart.

3D TAV, Sievers 0 BAV, and Sievers 1 BAV geometries were designed in SOLIDWORKS 2014 (Dassault Systems, Inc, Velizy-Villacoublay, France). Consistent with the measurements and models reported by Cao et. al., the TAV geometry consisted of three identical leaflets attached to a tri-lobed aortic root (Fig 1) [9]. To enhance computational stability, two straight-tube extensions (length: 12 mm) were added to the aortic root; one at the inlet section (ventricular extension) and one at the outlet section (aortic extension). The Sievers 0 BAV geometry consisted of two symmetric leaflets and sinuses. Sievers 1 BAV was modeled with one normal leaflet and a larger leaflet formed by fusion between that two remaining leaflets. Based on previous echocardiographic reports, the non-coronary leaflet and its sinus were modeled to span a circumferential angle of 120^o^ [10]. The two remaining leaflets were assumed to be identical and a raphe was placed along the free edge of these two leaflets. Creation of the fused leaflet were based on four anatomic landmarks in a manner described in [9].

### Finite-element simulations

The deployment of a self-expandable transcatheter aortic valve was modeled in the 3D TAV, Sievers 0 BAV, and Sievers 1 BAV models through finite element analyses. A high-fidelity model of the CoreValve Evolute R size 26 mm (Medtronic, Minneapolis, MN, USA) was discretized with 159,435 hexahedral linear reduced-integrated with hourglass control elements, 5,706 quadrilateral linear shell full-integrated elements and 32,388 triangular linear membrane elements for the metallic frame the pericardium leaflets and skirt, respectively. The pseudo-elastic NiTi material was modeled as a shape memory alloy and the pericardial material with a linear elastic law (Young’s modulus of 1 MPa and Poisson’s ratio of 0.45) [11,12]. The stent and the pericardium were fixed together with a node-to-node connection in the grids. A penalty self-contact was defined between the three leaflets. The post-implantation geometric orifice area (GOA) of each valve model was evaluated by projecting the free margin of the leaflets onto a plane parallel to the valve anulus (Fig 3).

The TAV, Sievers 0 BAV, and Sievers 1 BAV aortic roots were discretized with 23,040, 14,784 and 15,264 hexahedral linear full-integrated elements respectively, and the soft material were modeled with hyperelastic material based on Pasta et al. 2020 (C10 = 15KPa, C20 = 158 KPa) [7]. The TAV, Sievers 0 BAV, and Sievers 1 BAV valves were discretized with 67,362, 115,800 and 87,407 tetrahedral linear elements and the material modeled with hyperelastic materials based on Cao et al. (C10 = 32.823 KPa, C01 = 2.955KPa, C11 = 585.790KPa) [9]. Node-to-surface tied contacts were defined between the commissural edges of the native valves and the internal surface of the aorta. A penalty self-contact was defined between the leaflets themselves. As boundary conditions, both ends of the aorta were constrained in the longitudinal direction.

A mass-weighted damping (Rayleigh damping coefficient alpha) was set to 0.1 ms^-1^ for the pericardium parts of the device, 0.01 ms^-1^ for the stent, and 0.1 ms^-1^ for the aorta and native valve. To create the space to insert the catheter with the crimped device in the TAV, Sievers 0 BAV, and Sievers 1 BAV, a pre-dilatation of the valve was carried out within a rigid catheter. To follow the rigid catheter was slowly lifted from the annulus to the ascending aorta to deploy the device coaxially to the aorta. A selective mass-scaling was adapted to keep the time-step at a constant 10^−6^ s during the simulations.

Technical details, including mesh sensitivity analysis, damping coefficients, mass scaling, contact algorithms and steps of valve deployment are described in more detail in [13] and [14]. The simulations were performed by means of the finite element solver LS-DYNA R11 (ANSYS, Canonsburg, PE, USA), while pre- and post-processing with the BETA CAE Systems package v20.1 (BETA CAE Systems, Root, Switzerland) [13].

## Results

**Fig 2:**
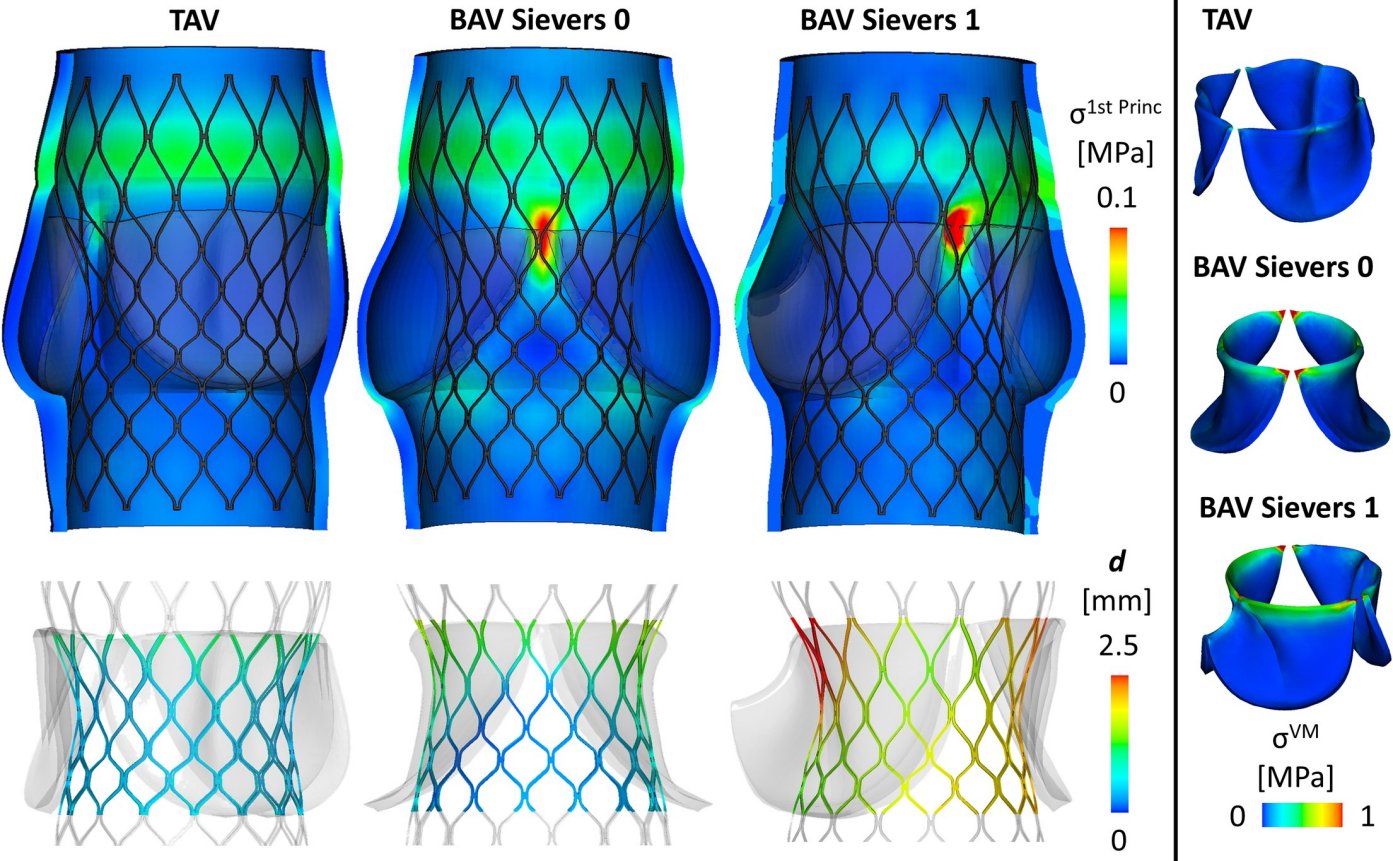
TAVR expansion in BAV subtypes, according to the Sievers classification, compared with TAV. Though Sievers 0 BAV leaflets experience higher Von Mises stress (σ^VM^) than Sievers 1 or TAV leaflets (6.64 MPa vs 4.19 MPa vs Tricuspid 0.55 MPa), stress concentration at the level of the commissures is greater in Sievers 1 BAV (0.316 MPa), than Sievers 0 (0.137 MPa) or TAV (0.056 MPa). Stent displacement, describing the physical deviation of the implanted device as compared to full unrestrained expansion, follows with stress (TAV 0.77 mm, Sievers 0 1.52 mm, Sievers 1 2.49 mm).

**Fig 3:**
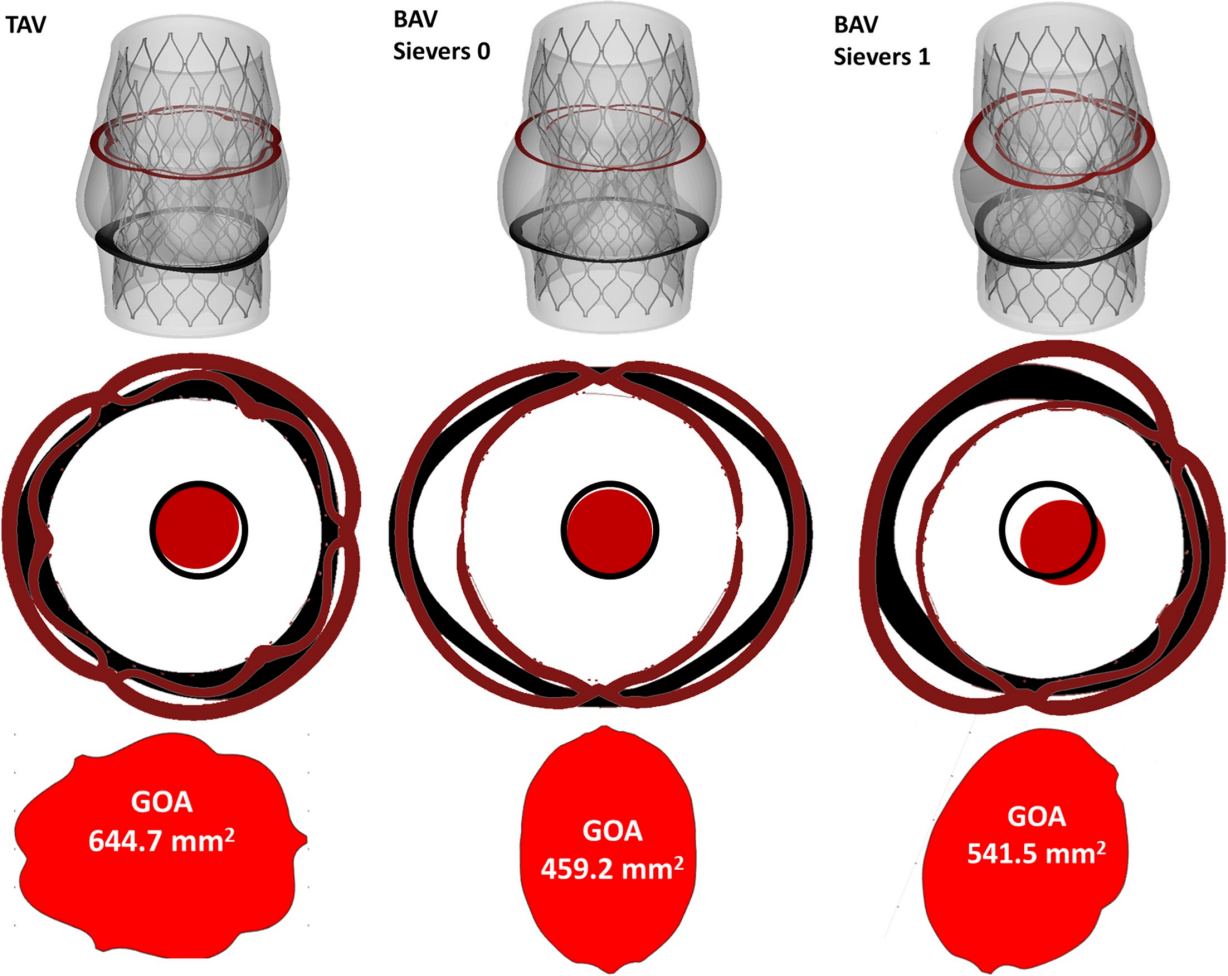
AVR expansion in BAV subtypes, according to the Sievers classification, compared with TAV. Device axisymmetry is noted by comparison of cross sections taken at the valve annulus (black) and leaflet free edge (red). In TAV and Sievers 0 BAV, device centroid at the annulus (black circle) shows little displacement from the device centroid measured at the leaflet free edge (red circle) (TAV: 0.33 mm, Sievers 0 BAV: 0.23 mm). In Sievers 1 BAV there is marked device eccentricity with 1.09 mm of displacement between the device centroid measured at the annulus and leaflet free edge. Devices in TAV and BAV experience different degrees of opening, with BAV deployment experiencing smaller geometric orifice areas (GOA), illustrated in cross section at the level of the leaflet free edge (TAV 644.68 mm^2^, BAV Sievers 0 459.18 mm^2^, BAV Sievers 1 541.55 mm^2^).

Post-TAVR states for TAV and the two BAV phenotypes differed by variations in valve strain at the aortic commissures, principle stress, implanted device orientation, and final orifice area. By all metrics (Table 1 and Fig 2) TAVR configurations in TAV was superior to BAV morphologies. Valve leaflet von Mises stress at the aortic commissures differed by valve configuration over a ten-fold range (TAV: 0.55 MPa, Sievers 0: 6.64 MPa, and Sievers 1: 4.19 MPa). First principle stress on the aortic wall was greater in Sievers 1 (0.316 MPa) and Sievers 0 BAV (0.137 MPa) compared to TAV (0.056 MPa). In addition to unfavorably high stresses seen in BAV compared to TAV, TAVR placement in Sievers 1 BAV demonstrated significantly asymmetric device alignment, with 1.09 mm of displacement between the center of the device measured at the annulus and at the leaflet free edge (Table 1 and Fig 3). This orifice displacement was marginal in TAV (0.33 mm) and even lower in Sievers 0 BAV (0.23 mm). Compared to ideal, unrestrained, expansion of the TAVR device, the TAVR stent frame in Sievers 1 BAV experienced a maximum strut deviation of 2.49 mm in contrast to 1.52 mm in Sievers 0 BAV and 0.77 mm in TAV. TAV allowed for the largest stent leaflet opening of implanted TAVR, with a maximum geometric orifice area (GOA) 644.68 mm^2^ compared to Sievers 0 (459.18 mm^2^) and Sievers 1 (541.55 mm^2^) BAV.

**Table 1:** Comparative stresses, strains, and resulting configuration experiences by both patient anatomy and TAVR devices implanted in Sievers 0 BAV, Sievers 1 BAV, and TAV.

Valve type	Aorta 1^st^ principle (MPa)	Leaflet Von Mises stress (MPa)	Leaflet opening (GOA mm^2^)	Stent displacement (mm)	Displacement of device centroid measured at the annulus and leaflet free edge (mm)
TAV	0.056	0.55	644.68	0.77	0.33
Sievers 0	0.137	6.64	459.18	1.52	0.23
Sievers 1	0.316	4.19	541.55	2.49	1.09

## Discussion

Although TAVR devices were designed for TAV AS, they have also been applied to BAV AS. BAV is an anatomically heterogeneous condition composed of several characteristic leaflet morphologies. Multiple studies have demonstrated limitations of TAVR in BAV compared to TAV including lower procedural success and greater residual moderate to severe paravalvular leak (PVL) and aortic root injury, conversion to open heart surgery, peri-procedural stroke, and permanent pacemaker (PPM) placement. The impact of valvular morphology on device durability remains unclear [15,16]. We sought to understand how BAV morphology correlates with and potentially drives the development of these complications mechanistically.

Consistent with other reports, our simulations demonstrate under expansion of TAVR devices within BAV, with important variations in the degree and character of this under expansion between BAV subtypes [3]. In our simulations, the Sievers 0 valve morphology imposes symmetric under expansion of TAVR devices, restricting expansion of the leaflet free edge by 28.7% compared to the same device deployed in TAV (see S1 Video). Although Sievers 0 BAV impairs full stent expansion, the TAVR device experiences symmetric radial expansion that retains axisymmetry similar to devices deployed in TAV (Fig 2). With minimal deviation of the device centroids, from annulus to leaflet free edge, devices deployed in Sievers 0 BAV are likely to allow for similar flow reconstitution compared to device deployed in TAV (Fig 3).

In contrast, the Sievers 1 morphology imposes asymmetric under expansion of TAVR devices. Though only 16% under expanded compared to TAV, device placement in this BAV configuration loses axisymmetry with three-fold greater device centroid displacement, between annulus and leaflet free edge, compared to both TAV and Sievers 0 and maximum strut deviation that is three-fold greater than devices deployed in TAV (Fig 3). Such deviations in expansion and alignment are thought to be associated with clinical complications [17–20]. Indeed, the loss of axisymmetry observed in devices deployed in Sievers 1 BAV, are unlikely to reconstitute normal flow and instead may impose eccentric jets that effect perfusion, which may manifest clinically as higher rates of stroke at 30 days compared to TAV [15].

Multiple reports have demonstrated that nearly a quarter of individuals receiving BAV TAVR require PPM with rates highest among self-expanding (SE) devices [15,21,22]. Our simulations reveal a concentration of stress in Sievers 1 BAV between the right and non-coronary cusp in exactly the region of the conduction band containing the left bundle branch (LBB). This stress concentration in Sievers 1 BAV TAVR is nearly six times as great as that seen in TAV (0.056 MPa vs 0.316 MPa). Interestingly, although Sievers 0 BAV experience a maximum stress nearly three times that of TAV, the stress concentration is not located near any sensitive conduction anatomy (Fig 2). Stratifying conduction abnormalities observed in BAV TAVR by Sievers subtype may lend predictive insights into rates of conduction abnormalities that could impact pre-TAVR screening and patient eligibility.

BAV TAVR, depending on the subtype involved, may encounter disparate combinations of device under expansion and asymmetry compared to TAV deployment. Although this study explores non-calcified native valves, the morphotype-dependent trends of device axisymmetry, tissue and device forces, as well as device under expansion are likely to be retained in calcified BAV given the histopathology of calcific valvular disease in BAV; in contrast to TAV, which often form nodular calcific lesions, the dominant form of calcification in BAV appears to be diffuse calcium deposition throughout the body of the valve leaflet, leading to valve stiffening [23]. BAV may encounter nodular lesions, most often occurring along sites of raphe, which would serve to exacerbate the morphotype-dependent trends of device expansion and tissue forces upon deployment demonstrated in our simulations [24]. Understanding the impacts of BAV morphology on patient-device relationships can help improve device selection, patient eligibility, and the overall safety of TAVR in BAV.

### Limitations

Our results should be interpreted in light of several limitations that accompany any such study and simulations. We employed idealized BAV and TAV geometries and our predictions should therefore be considered as illustrative of the extremes of differences generated by different morphologies. Patient-specific cases are likely to incorporate heterogeneities such as calcification and degeneration, which may impact device expansion and tissue forces upon deployment.

## Conclusion

It is increasingly important to understand TAVR performance especially in BAV geometries. We now show that morphology of AS results in dramatically different forces exerted on important regions, including the conduction zone, as well as significant variation in the degree and character of device expansion all of which may be implicated in complications reported in BAV TAVR. Further scrutiny should be applied to understand how these complications are distributed among the subtypes of BAV to proactively assess use of TAVR devices in BAV AS.

## Supporting information

S1 Video Device implantation in tricuspid (left) and bicuspid aortic valves with different subtypes of Sievers 0 (middle) and Sievers 1 (right).
(MP4)
